# Subcutaneous Phaeohyphomycosis Due to *Pyrenochaeta romeroi* Mimicking a Synovial Cyst

**DOI:** 10.3389/fmicb.2016.01405

**Published:** 2016-08-31

**Authors:** Aurélien Dinh, Bruno Levy, Frédérique Bouchand, Benjamin Davido, Clara Duran, Marin Cristi, Adrien Felter, Jérôme Salomon, Nawel Ait Ammar

**Affiliations:** ^1^Infectious Diseases Unit, Raymond Poincaré University HospitalGarches, France; ^2^Orthopaedic Surgery Unit, Ambroise Paré University HospitalBoulogne-Billancourt, France; ^3^Pharmacy Department, Raymond Poincaré University HospitalGarches, France; ^4^Pathology Unit, Ambroise Paré University HospitalBoulogne-Billancourt, France; ^5^Radiology Department, Raymond Poincaré University HospitalGarches, France; ^6^Institut Pasteur, Inserm UMR 1181Paris, France; ^7^Mycology and Parasitology Unit, Henri Mondor HospitalCréteil, France

**Keywords:** *Pyrenochaeta*, phaeohyphomycosis, fungal infection, surgical treatment, immunosuppression

## Abstract

Opportunistic subcutaneous fungal infections are increasing nowadays due to the growing number of medical conditions causing immunosuppression, especially organ transplant. The incidence rate of subcutaneous phaeohyphomycosis is very low. Most studies found are case reports. They showed a wide variation of clinical presentations. *Pyrenochaeta romeroi*, a fungus from the *Dematiaceae* group is a saprophyte found in soil and plants and a possible causative agent of phaeohyphomycosis. We present a rare case of subcutaneous phaeohyphomycosis caused by *P. romeroi* mimicking a synovial cyst in a diabetic patient.

## Introduction

Phaeohyphomycosis is a heterogeneous group of infections due to dematiaceous fungi with several clinical presentations, from superficial to deep infections (Borelli, 1979; Rinaldi, 1996). Subcutaneous phaeohyphomycosis cases are rare and clinical manifestations may vary (Vásquez-del-Mercado et al., 2013). One study found that the incidence rate of phaeohyphomycosis in a tertiary care center hospital varies from 1.0 to 3.1 cases per 100,000 patient-days (Ben-Ami et al., 2009). They affect predominantly the skin and can disseminate in immunocompromised patients, especially transplant patients.

There are more than 100 species of dematiaceous fungi associated with phaeohyphomycosis (*Alternaria* spp., *Curvularia* spp., and *Exophiala* spp…). There is no known correlation between etiologic agents and the clinical presentation of phaeohyphomycosis (Oliveira et al., 2016).

*Pyrenochaeta romeroi* has been recently described as a causative agent of phaeohyphomycotic cyst in few cases while it was already known as an agent of mycetoma (Thammayya et al., 1979; Girard et al., 2004; Badali et al., 2010a; Khan et al., 2011). This saprophytic fungus is widely distributed in the environment and found in soil, wood and plants in tropical area. It was first described by Borelli (1979).

The diagnosis of subcutaneous phaeohyphomycosis is difficult because of the lesions’ clinical polymorphism. It is usually established with histological examination and culture.

We present a case of subcutaneous phaeohyphomycosis due to *P. romeroi* mimicking a synovial cyst in a diabetic patient’s foot.

## Case Report

In April 2014, a 47-year-old woman, with a 2-year painful swelling on the inner edge of her right foot, was admitted to our hospital. She had no relevant medical history, except diabetes mellitus diagnosed 3 years before. She was born in Benin and had lived in France for 19 years. She was unemployed. Her last trip to Benin was 4 years ago and she stayed for a month. No notion of trauma was found.

Local examination revealed a soft to firm mass at the first radius of the right foot. It was slightly mobile, tender with normal overlying skin, and painful to pressure. The rest of clinical examination was normal. Biological investigations including hemogram, chemistry panel, C Reactive Protein level, liver enzyme level, serum protein electrophoresis, and serum immunoglobulin electrophoresis were all normal, except for blood glucose (413 g/dL). HIV serology was negative.

**FIGURE 1:**
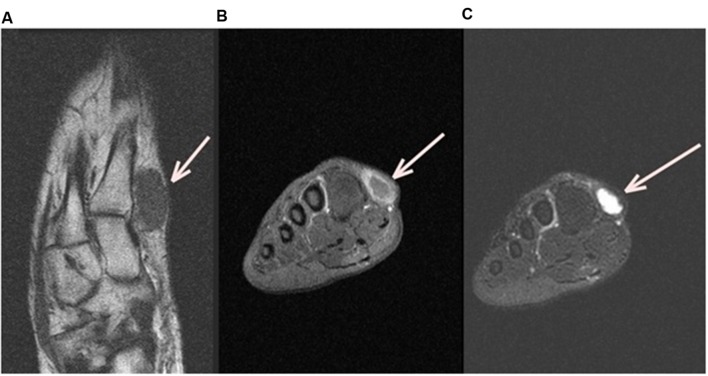
**Left Foot MRI (Magnetic Resonance Image).** Axial MRI T1 left foot image **(A)** shows a subcutaneous ovoid mass of 3cm × 2cm in contact with the first metatarse (white arrow), coronal T1 fat sat with gadolinium **(B)** and Coronal T2 fat sat **(C)** show, respectively, an hyposignal T1 and a hyper signal T2 with peripheric enhancement after injection.

A foot MRI (Magnetic Resonance Image) was realized. It showed a subcutaneous ovoid mass of 3cm × 2cm in contact with the first metatarse, associated to hyposignal T1 and hypersignal T2 with peripheric enhancement after injection (**Figure 1**), suggesting a diagnosis of synovial cyst.

A surgical treatment was performed. The cyst was incised and drained. As the patient presented no symptom, she had no antimicrobial prescription other than Amoxicillin clavulanate during 48 h. Patient was discharged 2 days after intervention without complications.

**FIGURE 2:**
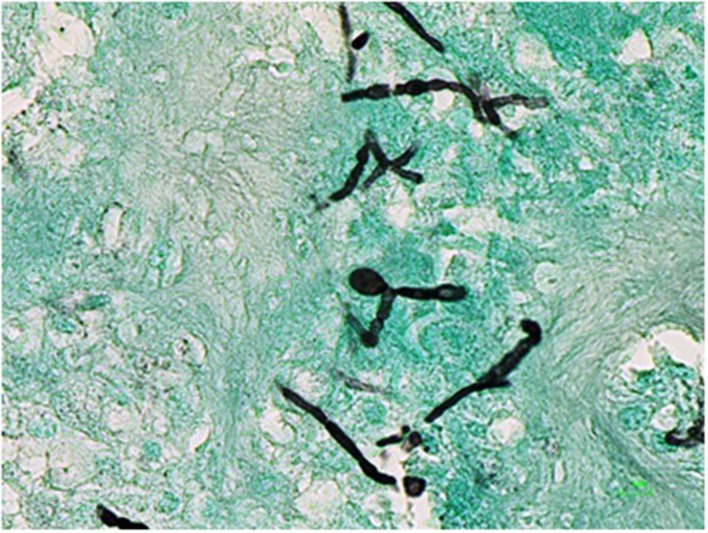
**Histological examination of the cyst on Grocott staining**.

**FIGURE 3:**
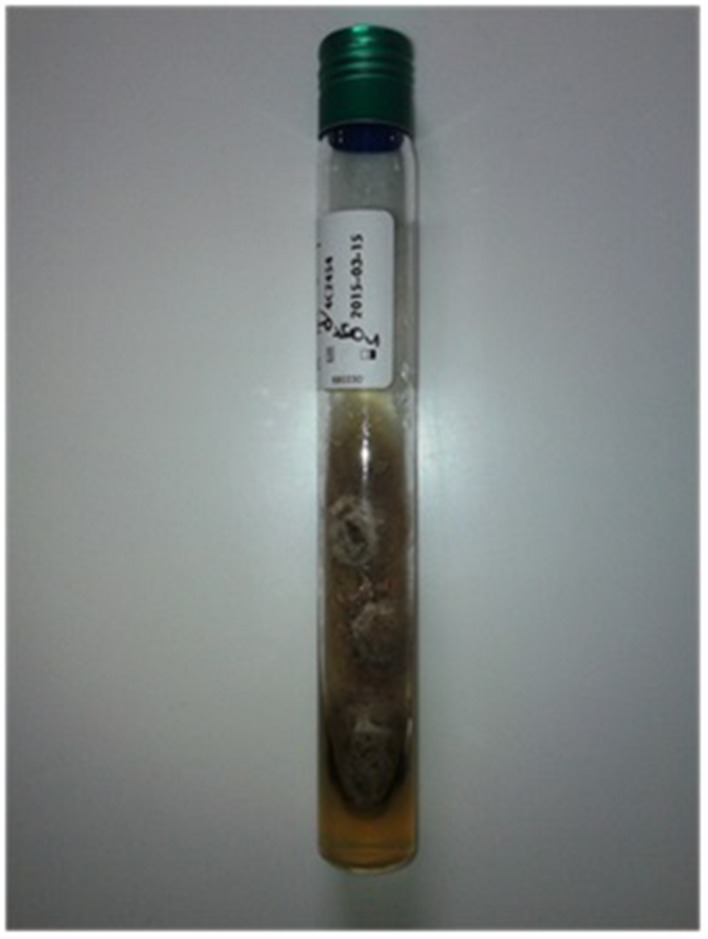
**Macroscopic aspect of *Pyrenochaeta romeroi* isolate on Sabouraud Chloramphenicol dextrose agar**.

**FIGURE 4:**
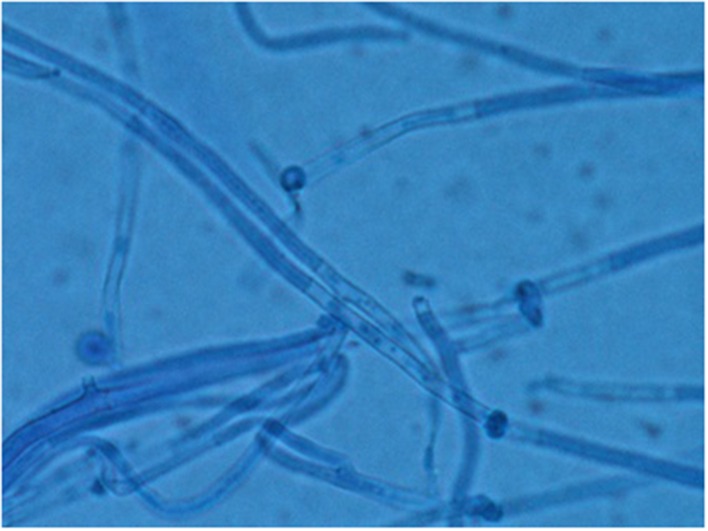
**Microscopic aspect of *P. romeroi* isolate (Blue cotton, ×40)**.

The cyst was sent for histological and microbiological analysis. Histology revealed a dense fibrous tissue associated with a granulomatous inflammation with abundant giant cells and a necrosis in which were found many irregular septate hyphae (**Figure 2**). Routine bacteriological cultures were negative. A dematiaceous mold grew in 10 days on Löwenstein–Jensen medium. A subculture on Sabouraud dextrose agar with Chloramphenicol was performed and incubated at 30°C. The macroscopic aspect of the culture was velvety and grayish (**Figure 3**). The microscopic aspect revealed septate and branched hyphae (**Figure 4**). No conidia were observed. On the basis of these microscopic and macroscopic examinations, the isolate was not identified.

Molecular identification was performed by PCR amplification and sequencing of internal transcribed spacer (ITS) regions. A homology for the obtained sequences was carried out using NCBI BLAST. The clinical isolate was identified as *P. romeroi*.

Antifungal MIC could not be determined due to the lack of growth on RPMI media.

Since the cyst’s surgical ablation was complete, no antifungal prescription was needed and the 2-year follow up did not show any relapse.

## Discussion

Subcutaneous phaeohyphomycosis often affect patients in immunosuppressive conditions, particularly kidney transplant and rheumatoid arthritis (Ronan et al., 1993). Its clinical spectrum is wide from local to systemic dissemination. *P. romeroi* is a saprophyte mostly found in soil or associated with plants, usually encountered in tropical and subtropical areas (Badali et al., 2010a; Khan et al., 2011). Agricultural workers have an increased risk of acquiring infection (Khan et al., 2011). In our case, the patient is a housewife but used to go in fields in Benin 4 years ago. The fungus is generally inoculated through direct traumatism by a plant or a soiled object. Though no history of trauma was found with our patient, foot localization might suggest this point of entrance for the fungus. Subcutaneous infections due to *P. romeroi* are rare (Girard et al., 2004; Badali et al., 2010a; Khan et al., 2011; Oliveira et al., 2016; Sharma et al., 2016). Cases are mostly related to immunosuppressive conditions (renal transplant, rheumatoid arthritis, corticosteroid…). Our patient only had diabetes mellitus. To our knowledge, this is the second case of subcutaneous phaeohyphomycosis due to *P. romeroi* described in a diabetic patient (Yadav et al., 2015).

The identification of *Pyrenochaeta* species is difficult (Badali et al., 2010a; Khan et al., 2011). Often pycnidia are not produced until 3 weeks on specific media. Thus, molecular identification is needed, using ITS sequencing as recommended by Santos et al. (2013). Based on a recent phylogenetic study, de Gruyter et al. (2009) renamed this fungus as *Medicopsis romeroi*.

No standard therapy is available for this infection. However, literature shows that surgery treatment has been successfully applied in some cases (Girard et al., 2004; Ferrer et al., 2009; Badali et al., 2010b; Verkley et al., 2010; Khan et al., 2011). The literature also suggests that antifungal susceptibility of *Pyrenochaeta* species is poorly documented due to low availability and low number of clinical isolates. Recently, ESCMID issued recommendations for treatment of phaeohyphomycosis. Surgery is the recommended first-line treatment, in addition to itraconazole or voriconazole to prevent any dissemination in immunocompromised patients (Chowdhary et al., 2014). Our patient showed signs of improvement after surgical treatment without any antifungal treatment.

## Concluding Remarks

We report an atypical case of subcutaneous phaeohyphomycosis due to *P. romeroi* mimicking a synovial cyst. This atypical presentation occurred in a non-immunosuppressed diabetic patient and healed without specific antimicrobial treatment. This presentation has not been reported before to the best of our knowledge.

## Ethics Statement

The patient signed a written consent form.

## Author Contributions

AD, BL, MC, AF, and NA substantially contributed to the conception of the work, the acquisition, analysis and interpretation of data and drafting and critically revising the work. FB, BD, CD, and JS substantially contributed to drafting and critically revising the work. All authors have final approval of the version to be published and agree to be accountable for all aspects of the work.

## Conflict of Interest Statement

The authors declare that the research was conducted in the absence of any commercial or financial relationships that could be construed as a potential conflict of interest.
